# Different Membrane Pathways Mediate Ca^2+^ Influx in Adrenal Chromaffin Cells Exposed to 150–400 ns Electric Pulses

**DOI:** 10.1155/2018/9046891

**Published:** 2018-03-29

**Authors:** Tarique R. Bagalkot, Robert C. Terhune, Normand Leblanc, Gale L. Craviso

**Affiliations:** Department of Pharmacology, University of Nevada, Reno School of Medicine, Reno, NV 89557, USA

## Abstract

Exposing adrenal chromaffin cells to 5 ns electric pulses (nsPEF) causes a rapid rise in intracellular Ca^2+^ ([Ca^2+^]_i_) that is solely the result of Ca^2+^ influx through voltage-gated Ca^2+^ channels (VGCCs). This study explored the effect of longer duration nsPEF on [Ca^2+^]_i_. Single 150, 200, or 400 ns pulses at 3.1 kV/cm evoked rapid increases in [Ca^2+^]_i_, the magnitude of which increased linearly with pulse width and electric field amplitude. Recovery of [Ca^2+^]_i_ to prestimulus levels was faster for 150 ns exposures. Regardless of pulse width, no rise in [Ca^2+^]_i_ occurred in the absence of extracellular Ca^2+^, indicating that the source of Ca^2+^ was from outside the cell. Ca^2+^ responses evoked by a 150 ns pulse were inhibited to varying degrees by *ω*-agatoxin IVA, *ω*-conotoxin GVIA, nitrendipine or nimodipine, antagonists of P/Q-, N-, and L-type VGCCs, respectively, and by 67% when all four types of VGCCs were blocked simultaneously. The remaining Ca^2+^ influx insensitive to VGCC inhibitors was attributed to plasma membrane nanoporation, which comprised the *E*-field sensitive component of the response. Both pathways of Ca^2+^ entry were inhibited by 200 *μ*M Cd^2+^. These results demonstrate that, in excitable chromaffin cells, single 150–400 ns pulses increased the permeability of the plasma membrane to Ca^2+^ in addition to causing Ca^2+^ influx via VGCCs.

## 1. Introduction

Calcium (Ca^2+^) is the universal intracellular signaling ion that plays a critical role in mediating a variety of cellular processes. It is not surprising, therefore, that a great deal of attention has been directed at developing techniques to manipulate intracellular Ca^2+^ levels ([Ca^2+^]_i_) in new ways. One such technique is the application of nanosecond electric pulses (nsPEFs) to biological cells.

Exposing cells to nsPEF causes an increase in [Ca^2+^]_i_ which, depending on the cell type and pulse parameters employed, can involve one or more mechanisms. On the one hand, numerous studies have reported that nsPEFs can trigger Ca^2+^ release from intracellular stores, in particular, the endoplasmic reticulum [1–5]. Most often this response has been attributed to the permeabilizing effect of the electric field on the organelle membrane that permits Ca^2+^ to be released into the cytoplasm [6]. In addition, nsPEFs have been reported to initiate phosphoinositide signaling that can also be involved in the observed rise in [Ca^2+^]_i_ [7]. On the other hand, nsPEF can evoke increases in [Ca^2+^]_i_ by causing Ca^2+^ influx across the plasma membrane. In this regard, two main pathways have been described. One is Ca^2+^ influx through Ca^2+^- permeable electropores (nanopores) that form in the lipid bilayer [8–12]. That is, the interaction of the electric field with the plasma membrane produces a direct conduit for Ca^2+^ to enter cells. The second pathway involves Ca^2+^ entry mediated by activation of ion channel proteins [13, 14].

A detailed characterization of the mechanisms underlying the manner in which nsPEFs cause a rise in [Ca^2+^]_i_ in particular cell types in conjunction with an understanding of the basis for differences in how various cell types undergo changes in [Ca^2+^]_i_ in response to nsPEFs is essential for developing nsPEF-based applications. Our specific goal has been to work toward the potential for nsPEF to serve as a novel electrostimulation modality for altering neural cell excitability. To this end we have been exploring the response of isolated adrenal chromaffin cells, a well-established nontransformed model of neural-type cells, to nsPEF in the low nanosecond regime (less than 10 ns in duration). In these cells, depolarizing stimuli cause Ca^2+^ influx via voltage-gated Ca^2+^ channels (VGCCs), which in turn triggers release of catecholamines by exocytosis [15]. Exposing chromaffin cells to a single 5 ns pulse applied at an amplitude of 5 MV/m evokes a similar neurosecretory response that is due to Ca^2+^ influx that occurs solely through VGCCs [13, 14, 16] with no detectable release of Ca^2+^ from internal stores [17, 18] and little or no direct influx of Ca^2+^ across the plasma membrane. The latter was established both in Ca^2+^ imaging experiments showing the absence of a rise in [Ca^2+^]_i_ when VGCCs were blocked [13, 14, 16] and in whole-cell patch clamp recordings in which Na^+^ was identified as the major ion that contributes to an instantaneous inward current (i.e., membrane permeabilization) evoked by the pulse [19]. In fact, Na^+^ influx via nanopores and the membrane depolarization that ensues are thought to be the underlying mechanism for nsPEF-evoked VGCC activation [14]. Thus, in this excitable neural-type cell, plasma membrane permeabilization resulting from exposure to a 5 ns pulse is quite distinctive in nature and consequence. These points take on added significance since there is no detectable uptake of YO-PRO-1 [17], a fluorescent dye used as an indicator of plasma membrane permeabilization in cells exposed to nsPEF. In addition, chromaffin cells do not undergo adverse morphological changes, such as cell swelling and cell blebbing [20] after exposure to one or multiple pulses.

Continuing to explore the potential use of nsPEF for modulating neural cell excitability, we describe here the results of studies in which fluorescence imaging of [Ca^2+^]_i_ was used to investigate Ca^2+^ responses in chromaffin cells exposed to single, longer duration nsPEF, specifically pulses having widths of 150, 200, or 400 ns. Such longer duration pulses may well have more profound effects on plasma membrane permeability which, from a physiological standpoint, would have important implications regarding chromaffin cell function and the potential use of such pulses for neuromodulation.

## 2. Materials and Methods

### 2.1. Chromaffin Cell Culturing and Preparation

Adrenal chromaffin cells were isolated by collagenase digestion of the medulla of fresh bovine adrenal glands obtained from a local slaughterhouse (Wolf Pack Meats, University of Nevada, Reno) and maintained in suspension culture in Ham's F-12 medium supplemented with 10% bovine calf serum, 100 U/ml penicillin, 100 *μ*g/ml streptomycin, 0.25 *μ*g/ml fungizone, and 6 *μ*g/ml cytosine arabinoside at 36.5°C under a humidified atmosphere of 5% CO_2_ as previously described [14, 16, 17]. Cells were used up until 14 days in culture. For experiments, large cell clusters were dissociated into single isolated cells with the protease dispase [21] and attached to fibronectin-coated 35 mm glass bottom dishes. Once attached, cells retained their spherical morphology and were used for a period not exceeding two days. Replicate experiments used cells from different cell preparations and different days in culture.

### 2.2. Fluorescence Imaging of Intracellular Ca^2+^ Levels

Cells were incubated with the cell-permeant Ca^2+^- sensitive fluorescent indicator Calcium Green-1-AM (1 *μ*M; Ex_480 nm_ and Em_535 nm_), for 45 min at 37°C in a balanced salt solution (BSS) containing 0.1% bovine serum albumin (BSA) (in mM): 145 NaCl, 5 KCl, 1.2 NaH_2_PO_4_, 2 CaCl_2_, 1.3 MgCl_2_, 10 glucose, and 15 HEPES, pH 7.4. After incubation, cells were washed twice with dye-free BSS lacking BSA and placed on the stage of a Nikon TE2000 epifluorescence microscope equipped with a 100x objective. For experiments conducted in the absence of extracellular Ca^2+^, the BSS lacked Ca^2+^ and contained 1 mM EGTA. Fluorescence images of the cells were captured before, during, and after stimulus application by an iXonEM + DU-897 EMCCD camera (Andor Technology, Ltd., Belfast, UK) using the open source microscopy software Micro-Manager (version 1.4, Vale Lab, UCSF, San Francisco, CA). The exposure time of the camera was set to 100 ms and images were captured at a rate of 7.5 Hz. Continuous baseline Ca^2+^ fluorescence of the cells was monitored 10 s prior to stimulus application and continued for 50 s after the stimulus. Sequences were analyzed using the public-domain image processing program ImageJ (https://imagej.nih.gov/ij/). The change in fluorescence intensity (Δ*F*) of the cells was calculated by subtracting the background fluorescence from the cell fluorescence (Δ*F* = *F*_cell_ − *F*_background_). Δ*F* was then normalized to the fluorescence intensity value (*F*_0_) at the time when the pulse was applied (Δ*F*/*F*_0_). Bright field images were obtained for each cell before and after the pulse.

### 2.3. nsPEF Exposure

Pulses were generated by a custom-designed high-voltage biphasic nanosecond pulse generator in which pulse widths ranged from 150, the lowest achievable under our conditions, to 1000 ns [22]. The system had two independent circuits that were designed to, respectively, deliver positive and negative phase pulses. The capacitors of each circuit were charged by independent high-voltage DC sources (Glassman High Voltage Inc., High Bridge, NJ) and connected to the pulse delivery electrodes through a high-speed, high-power MOSFET switch. In turn, each MOSFET switch was controlled by an external function generator (model 577-4C, Berkeley Nucleonics, San Rafael, CA) that had a separate channel for each phase of the pulse. Pulse magnitude was set by controlling the output of the voltage sources, and pulse durations and the interval between each phase of a biphasic pulse were set on the function generator. Both the voltage sources and the function generator were controlled by a custom LabVIEW program, and pulse traces were captured by an oscilloscope.

**Figure 1:**
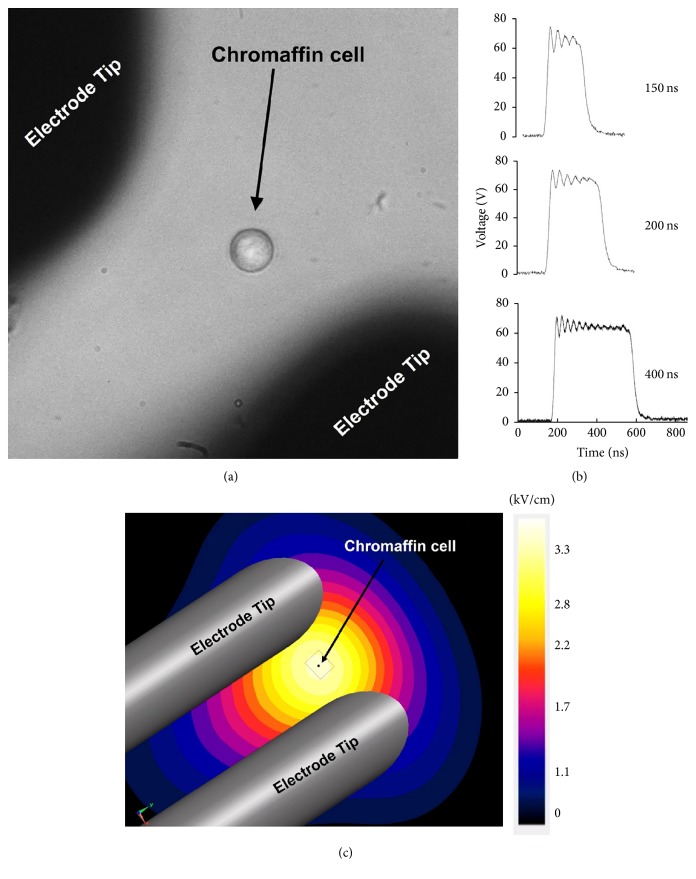
nsPEF exposure of chromaffin cells. (a) Photomicrograph of a chromaffin cell located at the center of the gap between the electrode tips. (b) Traces of a 150, 200, and 400 ns pulse (65 V applied to the electrodes to yield an *E*-field of 3.1/kVcm) captured by an oscilloscope. (c) Computed *E*-field distribution in the vicinity and at the location of an exposed cell. The dotted box represents the region over which a cell can be located in experiments.

In this study, cells were exposed only to unipolar pulses. Single pulses of 150, 200, and 400 ns were delivered to an attached chromaffin cell by means of two cylindrical tungsten rod electrodes (127 *μ*m diameter) with their tips separated by 100 *μ*m. Once immersed in the BSS bathing the cells, the electrode tips were positioned 40 *μ*m above the bottom of the dish using a motorized micromanipulator (model MP-225, Sutter Instruments, Novata, CA). The imaged cell was located at the center of the gap between the electrode tips (Figure 1(a)). Delivery of pulses was triggered externally by a LabVIEW program and a cell was exposed to a pulse only once. Traces for each pulse duration are shown in Figure 1(b). The 10–90% rise time was 14 ns for 150 ns pulses and 15 ns for both 200 ns and 400 ns pulses; the 10–90% fall time was 37 ns for 150 ns pulses and 39 ns for both 200 ns and 400 ns pulses. The *E*-field distribution in the vicinity and at the location of the target cell (Figure 1(c)) was computed using the commercially available Finite-Difference Time-Domain (FDTD) software package SEMCAD X (version 14.8.5, SPEAG, Zurich, Switzerland).

### 2.4. Statistical Analysis

Results are presented as the mean ± standard error (SE). Statistical analysis was done with SigmaPlot 12.5 software using either paired Student's *t*-test when the means of two groups were compared, or a one-way ANOVA test followed by Tukey post hoc multiple range tests for multiple group comparisons. *p* < 0.05 was considered statistically significant.

### 2.5. Reagents

Ham's F-12, dispase II, and the antibiotics antimycotics were obtained from Gibco Laboratories (Grand Island, NY, USA), bovine calf serum was purchased from Gemini Bio-products (West Sacramento, CA, USA), and collagenase B was obtained from Roche Diagnostics (Indianapolis, IN, USA). Calcium Green-1-AM was purchased from Invitrogen Corp. (Carlsbad, CA, USA), and *ω*-conotoxin GVIA and *ω*-agatoxin GIVA were purchased from Alomone Labs (Jerusalem, Israel). All other chemicals were reagent grade and purchased from standard commercial sources.

## 3. Results and Discussion

### 3.1. Single 150, 200, or 400 ns Pulses Caused a Rapid Increase in [Ca^2+^]_i_

**Figure 2:**
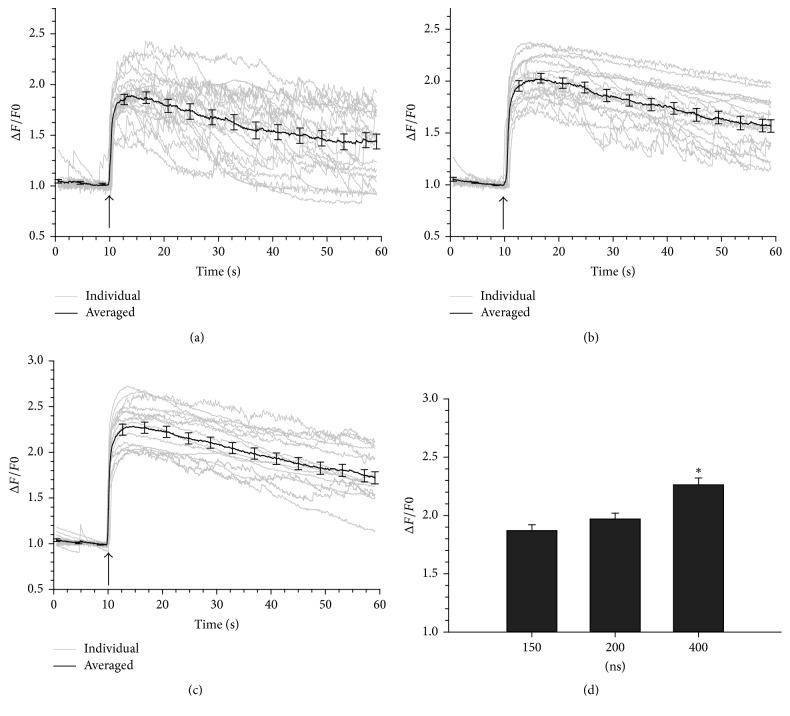
Rise in [Ca^2+^]_i_ evoked in chromaffin cells by nsPEF. Representative fluorescence traces together with the averaged responses for cells exposed to (a) a 150 ns pulse, (b) a 200 ns pulse, and (c) a 400 ns pulse. The *E*-field amplitude was 3.1 kV/cm and the arrow indicates when the pulse was delivered to the cells. The plot in (d) represents the average ± SE for the maximal increase in [Ca^2+^]_i_ for each pulse duration (150 ns, *n* = 20; 200 ns, *n* = 17; 400 ns, *n* = 16). ^*∗*^*p* < 0.05, significantly different from the 150 and 200 ns pulse.

In initial experiments, chromaffin cells were exposed to nsPEF comprising discrete pulse widths of 150, 200, or 400 ns. Pulses were applied at an *E*-field amplitude of 3.1 kV/cm. As shown in Figure 2(a), each pulse caused a rapid rise in [Ca^2+^]_i_ that was maximal after 1 to 2 s. Over the course of a 50 s monitoring period, [Ca^2+^]_i_ recovered to baseline in half the cells exposed to a 150 ns pulse but was more sustained and did not reach baseline levels in the remaining cells. A similar pattern of a more sustained rise in [Ca^2+^]_i_ elicited by nsPEF was also observed in cells exposed to a single 200 or 400 ns pulse. It is important note that the recovery of [Ca^2+^]_i_ to baseline in cells exposed to nsPEF, even pulses of a 5 ns duration, is much slower than in cells stimulated with the nicotinic receptor agonist 1,1-dimethyl-4-phenylpiperazinium [21] and the mixed nicotinic and muscarinic receptor agonist carbachol [18].

**Figure 3:**
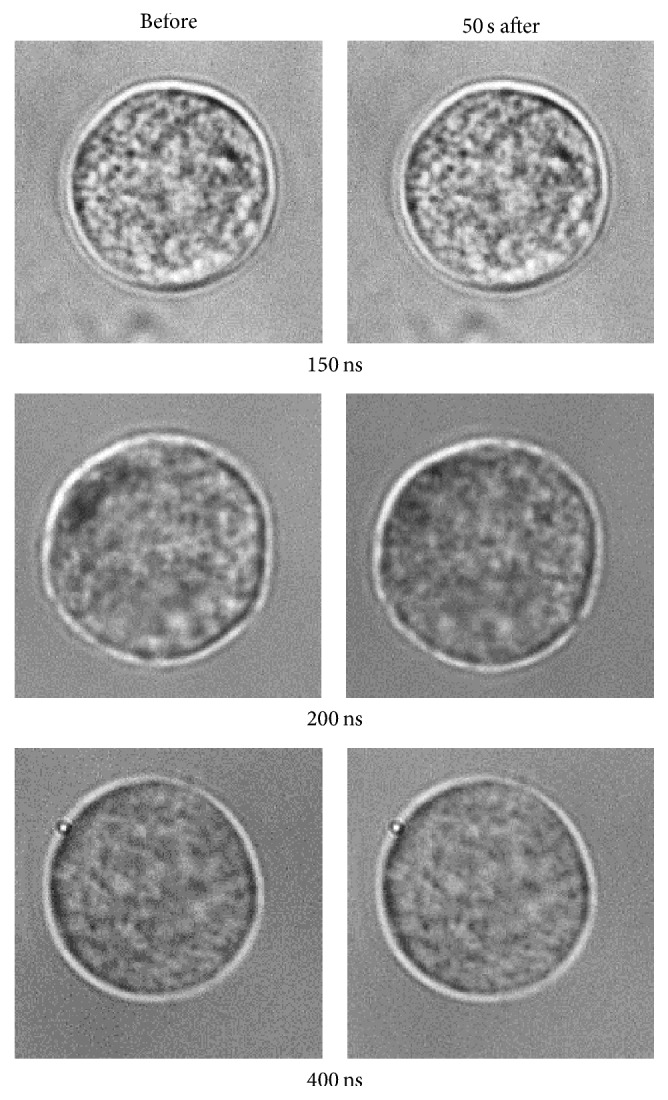
Representative bright field images of cells before and after exposure to a 150, 200, and 400 ns pulse. Each pulse was applied at an *E*-field of 3.1 kV/cm and images were captured before 0 s and 50 s after the pulse.

Figure 2(b) shows that the magnitude of the response increased with pulse duration. Post hoc analysis revealed a significant difference in the magnitude of the response for a 400 ns pulse compared with that for a 150 ns (*p* < 0.01) and 200 ns pulse (*p* < 0.01), but not for a 150 ns versus a 200 ns pulse (*p* = 0.379). Differences in the magnitude and duration of the Ca^2+^ response observed for each pulse width were not associated with differences in cell morphology even 50 s after nsPEF exposure (Figure 3).

**Figure 4:**
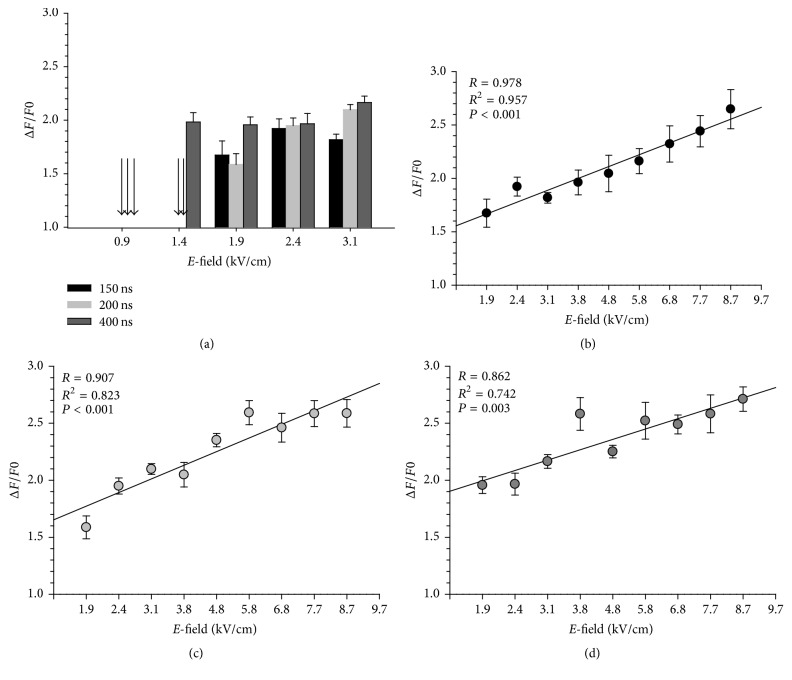
Peak Ca^2+^ responses evoked by a 150, 200, and 400 ns pulse at different *E*-field amplitudes. The plot in (a) represents the average ± SE for the maximal increase in [Ca^2+^]_i_ for each pulse duration at *E*-field amplitudes ranging from 0.9 to 3.1 kV/cm (*n* = 5–40). The arrows indicate the *E*-field amplitude at which there was no response. A regression analysis of peak Ca^2+^ responses for each pulse duration at *E*-field amplitudes ranging from 1.9 to 8.7 kV/cm is shown in (b) for a 150 ns pulse (*n* = 5–40); in (c) for a 200 ns pulse (*n* = 5–26); and in (d) for a 400 ns pulse (*n* = 5–15). All slopes in panels (b)–(d) were significantly different from 0.

The effect of pulse duration versus *E*-field amplitude was investigated next by exposing cells to a 150, 200, or 400 ns pulse at field strengths ranging from 0.9 to 8.7 kV/cm.  Figure 4(a) shows the peak Ca^2+^ responses elicited at the lower *E*-field amplitudes that were tested to determine the threshold. At 0.9 kV/cm, there was no detectable rise in [Ca^2+^]_i_ in cells exposed to either a 150, 200, or 400 ns pulse. At 1.4 kV/cm, only cells exposed to a 400 ns pulse exhibited a rise in [Ca^2+^]_i_, with 100% of the cells responding to the stimulus. At 1.9 kV/cm, increases in [Ca^2+^]_i_ were detected in 31% and 45% of the cells exposed to a 150 or 200 ns pulse, respectively. At 2.4 kV/cm or higher *E*-field amplitudes, all exposed cells responded to the pulses. Thus, the *E*-field threshold for evoking responses varied with pulse duration, with a slightly lower threshold for the longer duration pulses.

As shown in Figures 4(b)–4(d) for *E*-fields ranging from 2.4 to 8.7 kV/cm, linear regression analysis by least-square fitting of the datasets for pulse durations of 150, 200, and 400 ns revealed that the magnitude of the Ca^2+^ response elicited by nsPEF increased linearly with *E*-field amplitude (*p* < 0.05). This observation contrasts with a previous study from our group which showed that Ca^2+^ entry elicited by a 5 ns pulse above threshold was independent of *E*-field magnitude [18]. Although the mechanism underlying such differences is presently unknown and will require more investigation, the experiments described below will demonstrate that longer duration nsPEF elicited Ca^2+^ responses that were composed of at least two distinct Ca^2+^ entry pathways as opposed to 5 ns pulses in which Ca^2+^ entry occurred only via VGCCs [14]. Only one of these Ca^2+^ entry pathways was sensitive to *E*-field magnitude.

### 3.2. The Rise in [Ca^2+^]_i_ Required Extracellular Ca^2+^

**Figure 5:**
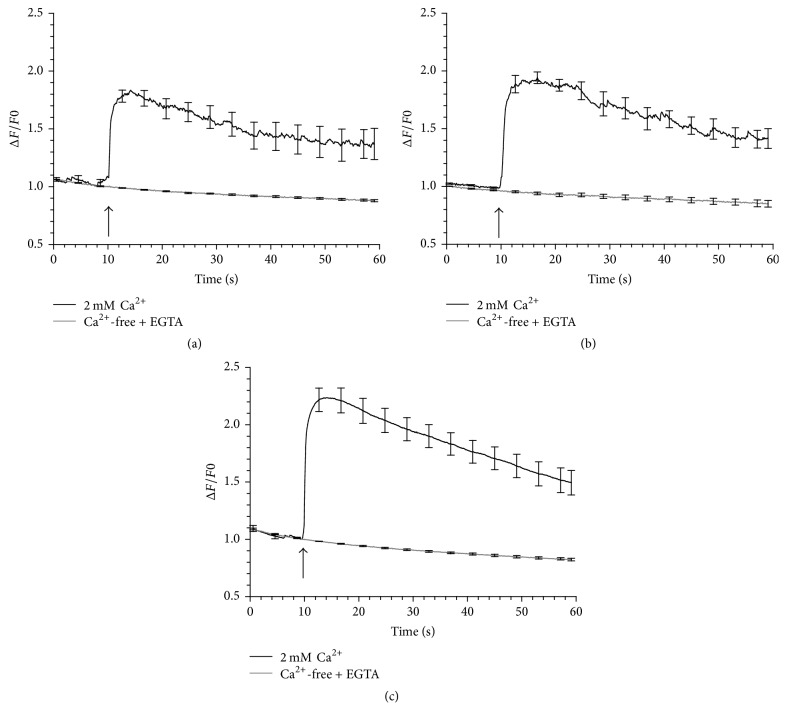
Effect of extracellular Ca^2+^ on the Ca^2+^ response of the cells to nsPEF. Averaged cell responses ± SE for cells exposed to (a) a 150 ns pulse, (*n* = 7–10), (b) a 200 ns pulse, (*n* = 6), and (c) a 400 ns pulse, (*n* = 10). Each pulse was applied in the presence and absence of extracellular Ca^2+^at an *E*-field of 3.1 kV/cm. The arrow indicates when the pulse was delivered to the cells.

To determine whether the source of Ca^2+^ responsible for nsPEF-evoked increases in [Ca^2+^]_i_ was intracellular or extracellular, cells were exposed to a 150, 200, or 400 ns pulse at 3.1 kV/cm in Ca^2+^-free BSS that contained EGTA. As shown in Figure 5, removal of extracellular Ca^2+^ abolished the response of the cells to nsPEF exposure for the three pulse durations tested. Thus, regardless of pulse duration, the mechanism by which 150–400 nsPEF exposure caused an increase in [Ca^2+^]_i_ in chromaffin cells at an *E*-field of 3.1 kV/cm, which is just above threshold for evoking a response (Figure 4(a)), was Ca^2+^ influx and not Ca^2+^ release from internal stores. This finding was not unexpected. As mentioned in the Introduction, exposing chromaffin cells to one or ten 5 ns pulses at an *E*-field of 5 MV/m, which is near the threshold value for evoking responses in this cell type, does not cause release of Ca^2+^ from intracellular stores [17, 18]. If shorter duration nanosecond pulses are more effective for causing release of Ca^2+^ from intracellular stores than longer duration pulses, as demonstrated by Semenov et al. (2013), and Ca^2+^ mobilization is not evoked in chromaffin cells exposed to 5 ns pulses, the ability of longer duration pulses to cause an intracellular permeabilizing effect is unlikely. Perhaps increasing the *E*-field amplitude of 150–400 ns pulses would lead to detectable Ca^2+^ mobilization from internal stores, as has been shown for cell exposures to 5 ns pulses [18]. Nevertheless, the results of this study unequivocally show that, for Ca^2+^ responses elicited by a stimulus just above threshold, the effects of longer duration nsPEFs are confined to the plasma membrane as they are for 5 ns pulses. We wish to point out however that, for any depolarizing stimulus that causes Ca^2+^ entry via VGCCs in chromaffin cells, there may be some calcium-induced calcium release (CICR) due to activation of ryanodine receptors as a consequence of the influx of Ca^2+^ [23]. We did not test for the presence of CICR since this would be a secondary effect unrelated to the pulse itself.

### 3.3. VGCCs Accounted for the Majority of Ca^2+^ Influx

**Figure 6:**
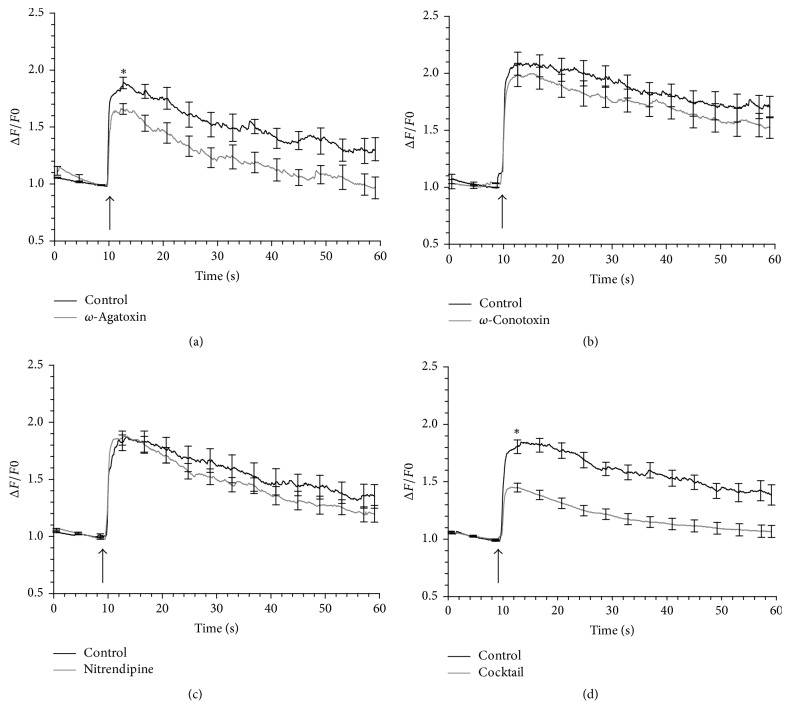
Effect of VGCC blockers on the Ca^2+^ response of the cells to a 150 ns pulse. Results are plotted as the averaged cell responses ± SE for cells exposed to a 3.1 kV/cm pulse in the absence and presence of (a) 100 nM *ω*-agatoxin IVA, (*n* = 6), (b) 20 nM *ω*-conotoxin GVIA, (*n* = 8–14) (c), 5 *μ*M nitrendipine, (*n* = 9–12), and (d) a cocktail of all three blockers, (*n* = 11–13). The arrow indicates when the pulse was delivered to the cells. Cell were pretreated with the blockers for 1 hr at room temperature. ^*∗*^*p* < 0.05, significantly different from control.

The involvement of VGCCs in mediating Ca^2+^ influx was studied in cells exposed to 150 ns pulses, which evoked Ca^2+^ responses that were abbreviated (Figure 2(a)) relative to longer duration nsPEFs (Figures 2(b) and 2(c)). For these studies, cells were exposed to a pulse at 3.1 kV/cm in the presence of blockers selective for P/Q-, N-, and L-type Ca^2+^ channels, all of which are expressed in bovine chromaffin cell [24]. Each blocker was used at the same concentration as that for assessing the role of the different types of VGCCs individually in mediating Ca^2+^ influx evoked by a 5 ns pulse and when combined in cocktail to block all VGCCs simultaneously. It was found that the cocktail caused complete inhibition of Ca^2+^ influx triggered by the pulse [14].  Figure 6 shows mean Ca^2+^ responses evoked by a 150 ns pulse in the presence or absence of each specific VGCC blocker (Figures 6(a), 6(b), and 6(c)) and in the presence or absence of a VGCC blocker cocktail that contained all three inhibitors (Figure 6(d)).  Table 1 provides a summary of the results.

The selective P/Q-type channel antagonist *ω*-agatoxin IVA (100 nM) reduced the magnitude of the Ca^2+^ response by 24% (*p* < 0.05), whereas the selective N-type channel antagonist *ω*-conotoxin GIVA (20 nM) reduced the magnitude of the rise in [Ca^2+^]_i_ by 9%, although the effect did not reach statistical significance. These results are comparable to those observed in cells exposed to 5 ns pulses [14]. However, blocking L-type channels with the dihydropyridine nimodipine (5 *μ*M) had no inhibitory effect, contrasting with a 50% reduction in Ca^2+^ influx when the stimulus was instead a 5 ns pulse [14]. Nitrendipine, another dihydropyridine blocker, was also without effect at 5 *μ*M. As a consequence, the extent to which the rise in [Ca^2+^]_i_ was reduced by the VGCC cocktail, 48% (*p* < 0.01), reflected primarily the combined inhibition of P/Q- and N-type channels. Thus, complete inhibition by the VGCC cocktail on Ca^2+^ influx evoked by a 150 ns pulse, as found for a 5 ns pulse, was not observed due to the lack of an inhibitory effect of dihydropyridines on L-type channels.

We next conducted experiments to further evaluate the contribution of VGCCs in meditating Ca^2+^ influx. In chromaffin cells, L-type channels comprise both Cav1.2 and Cav1.3 isoforms that are sensitive to dihydropyridines [25]. It is important to note that, depending on the strength of the stimulus applied to the cells, concentrations of dihydropyridines that completely block Cav1.2 channels may not be as effective for fully blocking Cav1.3 channels [26, 27]. As a consequence, nitrendipine has been used at higher concentrations to achieve full blockade of L-type channel mediated responses under these conditions [28]. For this reason, we elected to test the effect of increasing the concentration of nitrendipine fourfold (20 *μ*M). As shown in Table 1, nitrendipine at this higher concentration caused a 62% (*p* < 0.01) reduction in the Ca^2+^ response of the cells. The significant inhibitory effect now observed most likely reflected more effective blockade of L-type channels as well as partial blockade of other types of VGCCs, as has been described by other laboratories [29]. In support of this view, when cells were exposed to a 150 ns pulse in the presence of a cocktail of the VGCC inhibitors in which nitrendipine was present at 20 *μ*M, the extent to which the Ca^2+^ response was reduced was similar (67% versus 62%). We conclude from these results that VGCCs account for the majority of Ca^2+^ influx evoked by a 150 ns pulse and that the remaining ~35% of the response that is independent of VGCCs occurs via Ca^2+^- permeable nanopores. We also conclude that the requirement for a high concentration of dihydropyridines to block fully Ca^2+^ influx via L-type channels in cells exposed to a 150 ns pulse versus a 5 ns pulse indicates that the longer duration pulse serves as a much stronger cell stimulus, the basis for which is currently under investigation.

### 3.4. Ca^2+^ Influx Not Mediated by VGCCs Was *E*-field Sensitive

**Figure 7:**
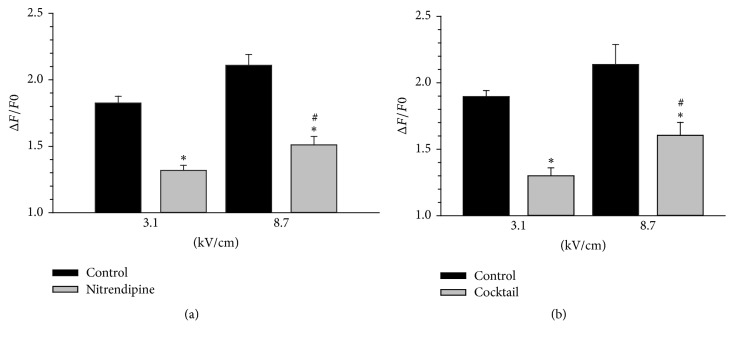
Peak Ca^2+^ responses in cells exposed to a 150 ns pulse at different *E*-field amplitudes when VGCCs were blocked. Results are plotted as the averaged cell responses ± SE for cells exposed to a 3.1 and 8.7 kV/cm pulse in absence and presence of (a) 20 *μ*M nitrendipine and (b) a cocktail of VGCCs containing 20 *μ*M nitrendipine (*n* = 15–25). Cells were pretreated with nitrendipine or the VGCC cocktail for 1 hr at room temperature. ^*∗*^*p* < 0.01, significantly different from the corresponding control. ^#^*p* < 0.01, significantly different from the 3.1 kV/cm treatment group.

VGCC activation is an all-or-none event. That is, once the membrane potential threshold for channel activation is reached, Ca^2+^ influx either will fully occur or will not occur at all. Thus, additional increases in stimulus strength will not lead to increases in the magnitude of Ca^2+^ entry due to VGCC activation, which has been shown for chromaffin cells exposed to 5 ns pulses [18]. This suggests that the *E*-field sensitive component of the Ca^2+^ response evoked by a 150 ns pulse (Figure 4) was most likely due to the membrane permeabilizing effect of the pulse and not VGCC activation. This was tested by exposing cells to a 150 ns pulse at 3.1 and 8.7 kV/cm in which VGCCs were blocked using 20 *μ*M nitrendipine alone or a cocktail of VGCC inhibitors that included 20 *μ*M nitrendipine. The results of these studies, shown in Figure 7, indicated that when VGCCs were blocked, the percentage of the remaining pulse-evoked increase in [Ca^2+^]_i_ was greater (39%) for the higher *E*-field amplitude of 8.7 kV/cm versus 3.1 kV/cm. That higher *E*-field strength having greater membrane permeabilizing effects is to be expected and help to explain the results presented in Figure 4.

### 3.5. Cd^2+^ Blocked Ca^2+^ Influx through Both VGCCs and Ca^2+^- Permeable Nanopores

**Figure 8:**
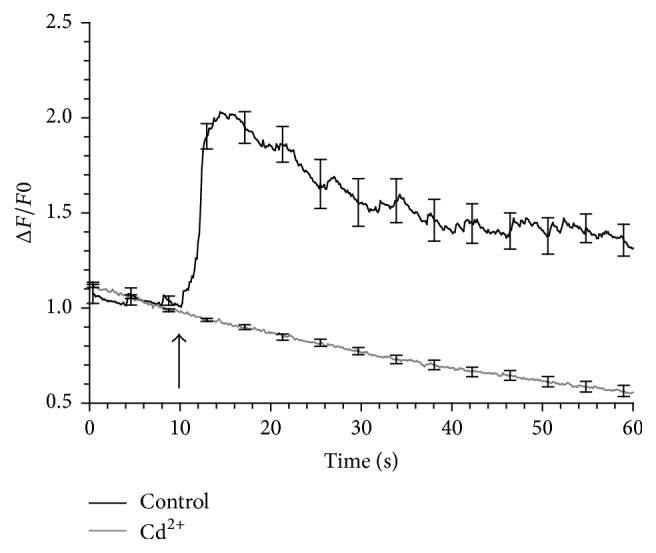
Effect of Cd^2+^ on the Ca^2+^ response of the cells to a 150 ns pulse. Results are plotted as the averaged cell responses ± SE for cells exposed to a 150 ns pulse at 3.1 kV/cm in the absence and presence of 200 *μ*M Cd^2+^. Cells were pretreated for 30 min with Cd^2+^ before exposure. The arrow indicates when the pulse was delivered to the cells (*n* = 8–11).

Cd^2+^ is an inorganic, nonselective blocker of VGCCs that was also used to evaluate the role of these channels in the Ca^2+^ response of the cells to 150 ns pulses. For this evaluation, cells were exposed to pulses in BSS containing 2 mM CaCl_2_ and 200 *μ*M CdCl_2_. As shown in Figure 8 and to our surprise, there was no rise in [Ca^2+^]_i_ in response to a 150 ns pulse when Cd^2+^ was present, indicating that this divalent ion not only blocked VGCCs but also Ca^2+^- permeable nanopores that were presumably formed in the plasma membrane. These results are interesting from two standpoints. First, Roth et al. (2013) reported that in mouse primary hippocampal neurons exposed to nsPEF, Cd^2+^ failed to prevent Ca^2+^ uptake into the cells due to plasma membrane permeabilization [30]. Pakhomov and Pakhomova (2010) similarly reported that plasma membrane conductance via nanopores was not blocked by Cd^2+^ [31]. Their studies instead showed that Gd^3+^ was a potent blocker of membrane conductance [32]. Second, while Cd^2+^ is known to bind to the same high affinity binding site for Ca^2+^ inside the VGCC pore and thus block Ca^2+^ entry [33, 34], it is unclear how Cd^2+^ can effectively compete with Ca^2+^ that is present at a tenfold higher concentration to block Ca^2+^ entry via a putative lipid nanopore. Future studies addressing this issue are needed to understand the basis for differences in plasma membrane conductance properties evoked by nsPEF in various cell types.

### 3.6. YO-PRO-1 Uptake into the Cells Was Detectable under Pulse Delivery Conditions That Caused Cell Swelling

**Figure 9:**
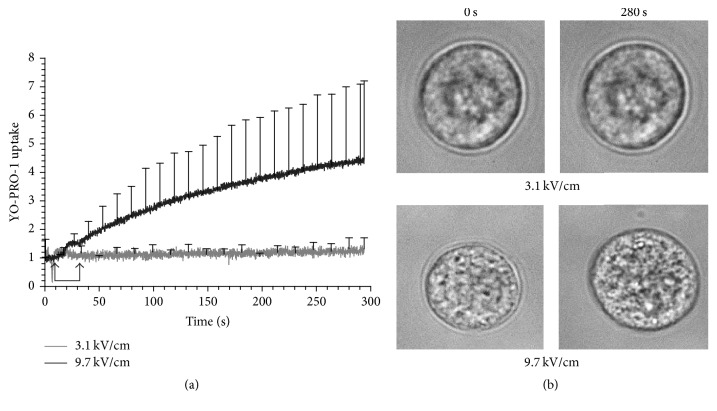
YO-PRO-1 uptake into cells exposed to 150 ns pulses. Cells were exposed to twenty, 150 ns pulses (1 Hz pulse repetition rate) in the presence of 2 *μ*M YO-PRO-1 at either 3.1 or 9.7 kV/cm. In (a) results are plotted as the averaged cell responses ± SE (3.1 kV/cm, *n* = 3 and 9.7 kV/cm, *n* = 4). Arrows indicate when the pulse train was delivered. In (b) representative bright field images of the cells before (0 s) and 280 s after the pulse train are shown.

The fluorescent dye YO-PRO-1 has been used as an indicator of plasma membrane permeabilization to nsPEF [35]. Interestingly, in chromaffin cells exposed to 5 ns pulses under conditions that cause membrane permeabilization [19], there is no detectable uptake of YO-PRO-1 into the cells [17], indicating that the electropores that were formed were too small to permit passage of the dye. In addition, as discussed in Introduction, the plasma membrane permeabilizing effects of the pulse are rather restrictive regarding the ionic species that enters the cells. That is, for reasons we do not yet understand, the membrane becomes permeable mainly to Na^+^ and not Ca^2+^ [19]. In the present study, we addressed the question of whether the plasma membrane permeabilizing effect of a longer duration (150 ns) pulse that results in Ca^2+^ influx would now allow YO-PRO-1 to be taken up by the cells. To address this question, 150 ns pulses were delivered to the cells in BSS containing 2 *μ*M YO-PRO-1. In cells exposed to a single pulse applied at 3.1 kV/m, YO-PRO-1 uptake was never observed (results not shown). Thus, even though the plasma membrane was now permeable to Ca^2+^, chromaffin cells were still resistant to YO-PRO-1 uptake, indicating that Ca^2+^ was entering the cells via nanopores that were still too small to permit YO-PRO-1 passage. Moreover, as shown in Figure 9(a), detectable uptake of the dye into the cells was not observed even in response to 20 pulses. However, when the *E*-field was increased to 9.7 kV/cm, 20 pulses caused YO-PRO-1 uptake that was accompanied by significant cell swelling (Figure 9(b)). Thus, for chromaffin cells, only extreme 150 ns exposure conditions will result in increased plasma membrane permeabilization to the extent where a dye typically used to detect and monitor permeabilization of the plasma membrane to nsPEF can enter the cells [36].

### 3.7. Conclusions

A hallmark feature of cells exposed to nsPEFs is the formation of nanometer-size pores in the plasma membrane that, in many cell types, renders the membrane permeable to Ca^2+^. In isolated adrenal chromaffin cells exposed to 5 ns pulses, the plasma membrane does not become permeable to Ca^2+^ and influx of Ca^2+^ occurs only via VGCCs. However, increasing pulse duration to 150 ns causes significant Ca^2+^ influx via plasma membrane nanopores in addition to Ca^2+^ influx through VGCCs. Nevertheless, regardless of whether the enhanced permeabilizing effect is the result of larger pores or a different structure of the pores due to the longer application of the electric field, the cells still did not take up YO-PRO-1 unless they were exposed to a large number of nsPEFs at a high electric field intensity that causes significant cell swelling. Thus, pulse duration and amplitude, which do not affect VGCC-mediated Ca^2+^ influx, that is, an all-or-none response, appear to selectively modify membrane conductance to ions in this cell type in a manner that still limits YO-PRO-1 uptake. Taken together, our data suggest that neurosecretion could be fine-tuned by modulating the Ca^2+^ response through manipulation of nsPEF amplitude and duration. Studies examining these possibilities are underway.

## Figures and Tables

**Table 1:** Effect of blocking VGCCs on 150 ns pulse-induced increases in [Ca^2+^]_i_.

VGCC blocker	Relative fluorescence intensity (Δ*F*/*Fo*)		Reduction in amplitude (%)	*t*- test (*p* value)
	Control	VGCC blocked		
*ω*-Agatoxin GVIA (100 nM)	1.85 ± 0.06	1.64 ± 0.05	24	*p* < 0.05
*ω*-Conotoxin (20 nM)	2.06 ± 0.09	1.97 ± 0.05	9	NS
Nimodipine (5 *μ*M)	1.69 ± 0.04	1.69 ± 0.09	0	
Nitrendipine (5 *μ*M)	1.85 ± 0.08	1.88 ± 0.05	0	
Cocktail^*∗*^	1.82 ± 0.05	1.43 ± 0.03	48	*p* < 0.01
Nimodipine (20 *μ*M)	1.87 ± 0.07	1.41 ± 0.07	53	*p* < 0.01
Nitrendipine (20 *μ*M)	1.82 ± 0.05	1.31 ± 0.03	62	*p* < 0.01
Cocktail^*∗∗*^	1.89 ± 0.04	1.29 ± 0.05	67	*p* < 0.01

^*∗*^Nitrendipine at 5 *μ*M; ^*∗∗*^nitrendipine at 20 *μ*M; NS: not significant.
